# Cardiac Structure and Function in Patients With Obesity and Non-alcoholic Fatty Liver Disease

**DOI:** 10.7759/cureus.43711

**Published:** 2023-08-18

**Authors:** Zemfira Asatullina, Albina V Sineglazova

**Affiliations:** 1 Primary Care and General Practice, Kazan State Medical University, Kazan, RUS

**Keywords:** nafld and obesity, nonalcoholic fatty liver disease (nafld), transthoracic echocardiography (tte), heart failure with preserved ejection fraction (hfpef), cardiometabolic risk, cardiometabolic diseases

## Abstract

Introduction

Non-alcoholic fatty liver disease (NAFLD) has emerged as a leading cause of chronic liver disease worldwide. The global prevalence of NAFLD is expected to increase dramatically with the increasing prevalence of obesity and type 2 diabetes mellitus (T2DM). The role of NAFLD as a cardiometabolic risk factor or component of metabolic syndrome on the heart remains unclear. Thus, the independent effect of NAFLD on structural and functional heart parameters warrants validation. Our goal was to study cardiac structure and function in subjects with obesity and NAFLD.

Methods

A total of 164 patients were examined in this cross-sectional study. Participants were grouped based on BMI and the presence or absence of abdominal obesity (AO) and/or NAFLD. The subjects were divided into four groups: group 1: normal BMI without AO and NAFLD; group 2: BMI ≥ 25 kg/m^2^ or AO without NAFLD; group 3: BMI ≥ 25 kg/m^2^ and AO without NAFLD; group 4: patients with BMI ≥ 25 kg/m^2^, AO, and NAFLD. We performed a thorough clinical examination, a biochemical blood analysis, and echocardiography. Indices of liver steatosis and fibrosis were calculated. A study of liver assessment of the controlled attenuation parameter (CAP) and liver stiffness measurement (LSM) was conducted. Statistical analysis was performed using IBM SPSS Statistics version 26 (IBM Corp., Armonk, NY).

Results

The mean age of the participants was 35.0 (31.0-38.5) years. We found a higher frequency of multiple cardiometabolic risk factors in the general cohort. However, on comparing groups 3 and 4, we found a higher frequency of hyperinsulinemia, homeostatic model assessment of insulin resistance (HOMA-IR), and obesity (p < 0.05). To assess the role of NAFLD independent of obesity, we conducted further analyses after adjusting for BMI. Among patients with NAFLD, we observed a similar trend for parameters of carbohydrate metabolism (p < 0.005). In individuals with NAFLD, an increase in left atrial (LA) volume, interventricular septal (IVS) thickness, and left ventricular (LV) myocardial mass, and a decrease in LV ejection fraction and LV stroke volume index were found (p < 0.005). The hepatic steatosis index (HSI) correlated with LA volume, LV end-systolic volume (ESV) and LV end-diastolic volume (EDV), stroke volume, and LV myocardial mass. An association between an increase in CAP score and an increase in the LA volume, stroke volume index, IVS thickness, LV myocardial mass, and the values of LSM with an increase in the LA volume was established.

Conclusion

The presence of NAFLD without cardiovascular disease and diabetes mellitus revealed an association with the structural and functional parameters of the heart. The results of this study can also be used to improve the effectiveness of a comprehensive assessment of patients and to develop strategies for the primary and secondary prevention of heart failure with preserved ejection fraction in NAFLD.

## Introduction

Over the last few years, non-alcoholic fatty liver disease (NAFLD) has emerged as a leading cause of chronic liver disease worldwide, affecting up to 30% of the world’s adult population [1,2]. The estimated global prevalence of NAFLD is projected to increase from 25% to 178% by 2030, owing to the increasing prevalence of obesity and type 2 diabetes mellitus (T2DM) [2]. NAFLD promotes the progression of coronary atherosclerosis and affects cardiac structures, thereby increasing the risk of cardiac hypertrophy, which may lead to new-onset heart failure (HF) [3-5]. These may be due to the role of one or several factors, such as pro-inflammatory factors (e.g., interleukin (IL)-1β, IL-6, tumor necrosis factor, and other cytokines), insulin resistance and related disorders, activated renin-angiotensin-aldosterone system, and vasoactive and thrombogenic molecules (e.g., plasminogen activator inhibitor 1 and tumor growth factor β) [3]. A meta-analysis of 16 cross-sectional studies (n = 32,000) found that NAFLD was associated with subclinical myocardial alterations (increased left ventricular (LV) mass), as well as lower early diastolic relaxation (e’) velocity, higher LV filling pressure, and larger left atrial volume [6]. Simon et al. demonstrated that NAFLD was associated with an approximately 65% increased risk of incident major adverse cardiovascular events (MACE, defined as nonfatal coronary heart disease (CHD), stroke, HF, or cardiovascular death) over a median of 13.6 years [7]. This risk was independent of cardiometabolic risk factors and progressively increased with worsening liver disease severity. Furthermore, the risk of each component of MACE (including new-onset HF) increased across all NAFLD histological categories [7]. In the above studies, patients with diabetes mellitus were also considered; hence, it is difficult to judge an independent relationship. However, other authors believe that a relationship between NAFLD, metabolic syndrome, and insulin resistance has been established. Thus, the independent effect of NAFLD on structural and functional heart parameters and cardiovascular disease progression warrants validation [8,9]. Our goal was to study the features of cardiac structure and function in subjects with obesity and NAFLD.

## Materials and methods

Study setting and participants

This cross-sectional study was conducted at the Consultative Diagnostic Center of Aviastroytelniy District of Kazan, affiliated with the Department of Primary Care and General Practice of Kazan State Medical University. Participants were selected according to their body mass index (BMI) (normal weight, overweight, and obese) in accordance with the inclusion and exclusion criteria. The sample size was calculated using the Epi Info^TM^ application 5.5.5. (CDC, Atlanta, GA) for iOS 14.4.1. In total, 164 (female: 84; male: 80) individuals aged 25 to 44 years, with a median age of 35.0 (31.0-38.5) years, were included in the study.

Inclusion criteria

The inclusion criteria were individuals aged 25-44 years who provided voluntary informed consent to participate in this study.

Exclusion criteria

The exclusion criteria included refusal of the subject to participate in the study; patients with mental illness hampering the interview; the presence of verified cardiometabolic diseases (T2DM, coronary artery disease, HF, atrial fibrillation, chronic kidney disease); antiphospholipid syndrome and autoimmune inflammatory diseases; the presence of verified oncology; decompensatory states of concomitant diseases or conditions (liver disease including but not limited to viral hepatitis, alcoholic liver disease, kidney disease, etc.), acute infectious diseases, diseases of the endocrine system, and other diseases and conditions that are secondary causes of obesity; medical implants including a pacemaker, silicone implants, and metal prostheses; and pregnant and lactating women.

Clinical and biochemical data collection

The clinical assessment included detailed patient history, physical examination, and anthropometry. The World Health Organization classification was used to estimate the BMI [10]. Abdominal obesity (AO) was defined as a waist circumference (WC) ≥ 94 cm in men and ≥ 80 cm in women and/or a waist-to-hip ratio (WHR) greater than 0.9 in men or 0.85 in women [11]. Body composition was assessed using a body composition monitor (Tanita BC-601, Tokyo, Japan). A visceral fat rating of 1-12 was considered normal, and 13-59 was defined as an excess visceral fat level [12]. Blood pressure was measured at rest according to guidelines [13].

All biochemical blood analyses were performed using fasting venous blood samples at a certified laboratory. The lipid profile (total cholesterol, triglycerides, high-density lipoprotein cholesterol (HDL-c), low-density lipoprotein cholesterol (LDL-c), and non-HDL-c), carbohydrate metabolism (fasting plasma glucose, oral glucose tolerance test, glycated hemoglobin, and insulin), liver function (alanine aminotransferase (ALT) and aspartate aminotransferase (AST)), and uric acid levels were analyzed on a Beckman Coulter automated analyzer AU480 (Beckman Coulter Inc., Brea, CA) using Beckmann Coulter assays.

Echocardiographic investigation

Echocardiography was performed on a Mindray DC-8 machine (Mindray Medical International Limited, Shenzhen, China) in M- and B-modes with tissue Doppler imaging per the guidelines [14]. We evaluated the volume and size of the left atrium (LA), end-diastolic volume (EDV), end-systolic volume (ESV), ejection fraction (EF), stroke volume (SV), SV index, EDV index, ESV index, end-systolic dimension (ESD), cardiac output (CO), interventricular septal thickness (IVS), relative wall thickness (RWT), left ventricular mass, left ventricular mass index (LVMI), and left ventricular posterior wall thickness (PWT).

Ultrasound investigation

Ultrasound examination was conducted using a Mindray DC-8 (Mindray Medical International Limited, Shenzhen, China). NAFLD was diagnosed based on the results of a transabdominal ultrasound examination of the liver [15].

Upon diagnosis of NAFLD, the following liver steatosis and fibrosis indices were calculated: non-alcoholic fatty liver disease-liver fat score (NAFLD-LFS), hepatic steatosis index (HSI), triglyceride-glucose (TyG) index, and the fibrosis-4 (FIB-4) liver fibrosis index. Individuals with higher indices further underwent a study of controlled attenuation parameter (CAP) and liver stiffness measurement (LSM) using a FibroScan machine (Echosens, France) (n = 17).

Grouping of patients

Participants were grouped based on BMI and the presence or absence of AO and/or NAFLD. Participants were divided into four groups: group 1: normal BMI without AO and NAFLD; group 2: BMI ≥ 25 kg/m^2^ or AO without NAFLD; group 3: BMI ≥ 25 kg/m^2^ and AO without NAFLD; group 4: BMI ≥ 25 kg/m2, AO, and NAFLD. The median age of the participants in groups 1-4 was 34.0 (30.0-38.0) years, 34.0 (28.0-37.0) years, 36.0 (32.0-39.0) years, and 38.0 (34.0-41.0) years, respectively.

Statistical analysis

Statistical analyses were performed using the IBM SPSS® Statistics version 26 (IBM Corp., Armonk, NY). The normality of continuous variables was tested using the Kolmogorov-Smirnov test. Since the data distribution was non-normal, non-parametric analytical methods were used. Continuous variables were presented as a median and interquartile range (IQR, 25th-75th percentile). Descriptive statistics were used for the categorical variables. Statistical differences in categorical variables were tested using Pearson's chi-square test. The Mann-Whitney U-test was used to compare two independent groups, and the Kruskal-Wallis test was used to compare three or more groups. The area under the curve (AUC) of the receiver operator characteristic (ROC) curve was used. The AUC was calculated to assess changes in the structural and functional parameters of the heart. The sensitivity and specificity were calculated. The highest value of the sum of sensitivity and specificity in favor of sensitivity was used to determine cut-off values for detecting changes in hemodynamic parameters. Differences between the groups were considered statistically significant at p < 0.05.

Ethical approval

All individuals gave voluntary informed consent for inclusion before participating in the study. The study was conducted in accordance with the Helsinki Declaration (amended 2013), and the protocol was approved on June 22, 2021, by the Local Ethics Committee of Kazan State Medical University (meeting protocol number 6).

## Results

**Figure 1:**
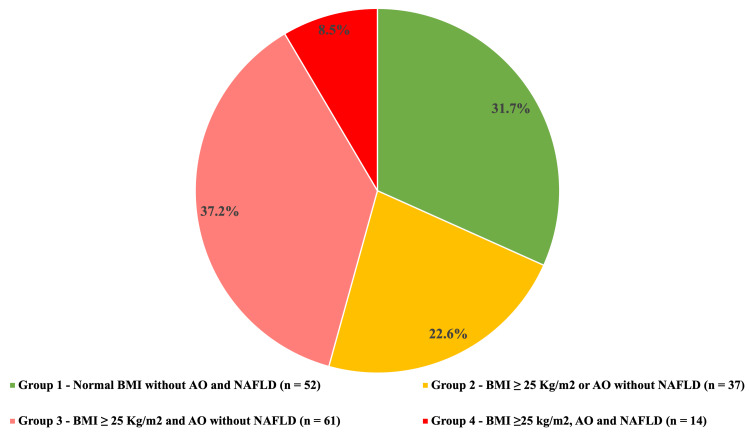
Distribution of study participants across various groups BMI: body mass index; AO: abdominal obesity; NAFLD: non-alcoholic fatty liver disease.

In the general cohort, more than half of the patients (n = 106; 64.6%) had a BMI ≥ 25 kg/m^2^, including 50 patients (30.4%) with general obesity. AO was observed in 50.6% (n = 83) of the patients. Although all participants were young, subjects from groups 1 and 2 were significantly younger than those from group 4 (p < 0.05). The gender distribution in the groups was comparable (p = 0.213-1.000). The patient distribution across various groups is shown in Figure 1. One-third of the studied individuals did not show an increase in BMI, AO, or NAFLD. NAFLD was diagnosed in only 8.5% of subjects (n = 14).

The detailed characteristics of the cardiometabolic risk factors in the studied group are presented in Table 1. The frequency of hypercholesterolemia differed across all groups except groups 3 and 4. A similar trend was noted for the increase in LDL-c and non-HDL-c levels. Prediabetes was less frequent in group 1 than in groups 2, 3, and 4. In contrast, the frequency of insulin resistance increased from group 1 to group 4. Hyperinsulinemia was found more often in group 4 than in groups 1, 2, and 3. Elevated blood pressure was less frequent in group 1 than in groups 2, 3, and 4. The frequency of obesity was significantly lower in group 2 than in groups 3 and 4, and group 3 than in group 4. AO was less common in group 2 when compared with groups 3 and 4, in which it was 100%. Excess visceral fat level was seen more often in group 4 compared with groups 2 and 3.

**Table 1:** Cardiometabolic risk factors in people with obesity and NAFLD Note: n: number of participants; %: proportion of subjects presented as percent; p_1,2_: significance between group 1 and group 2; p_1,3_: significance between group 1 and group 3; p_1,4_: significance between group 1 and group 4; p_2,3_: significance between group 2 and group 3; p_2,4_: significance between group 2 and group 4; p_3,4_: significance between group 3 and group 4, calculated using chi-squared test. BMI: body mass index; AO: abdominal obesity; NAFLD: non-alcoholic fatty liver disease; HDL-c: high-density lipoprotein cholesterol; LDL-c: low-density lipoprotein cholesterol; non-HDL-c: non-high-density lipoprotein cholesterol; HOMA-IR: homeostatic model assessment of insulin resistance; NC: not calculated.

Parameter	Normal BMI without AO and NAFLD (n = 52)	BMI ≥ 25 kg/m^2 ^or AO without NAFLD (n = 37)	BMI ≥ 25 kg/m^2 ^and AO without NAFLD (n = 61)	BMI ≥ 25 kg/m^2^, AO, and NAFLD (n = 14)	р_₁,₂_	р_₁,₃_	р_₁,₄_	р_₂,₃_	р_₂,₄_	р_₃,₄_
	1	2	3	4						
	n (%)	n (%)	n (%)	n (%)						
Total cholesterol ≥ 5 mmol/L	16 (30.8)	10 (27.0)	33 (54.1)	9 (64.3)	0.814	0.014	0.031	0.012	0.023	0.561
Triglycerides ≥ 1.7 mmol/L	3 (5.8)	4 (10.8)	16 (26.2)	5 (35.7)	0.443	0.005	0.008	0.076	0.093	0.517
Low HDL-c (in males < 1.0 mmol/L; in females < 1.2 mmol/L)	8 (15.4)	14 (37.8)	20 (32.8)	3 (21.4)	0.024	0.048	0.688	0.665	0.334	0.529
LDL-c > 3 mmol/L	23 (44.2)	15 (40.5)	43 (70.5)	13 (92.9)	0.829	0.007	0.002	0.006	0.001	0.100
Non-HDL-c > 3.4 mmol/L	20 (38.5)	15 (41.7)	41 (67.2)	12 (85.7)	0.826	0.003	0.002	0.019	0.010	0.210
Hyperuricemia	4 (7.7)	6 (16.2)	8 (13.3)	0 (0)	0.308	0.377	0.571	0.770	0.170	0.339
Prediabetes	3 (5.8)	8 (21.6)	15 (24.6)	6 (42.9)	0.046	0.009	0.002	0.809	0.166	0.196
HOMA-IR > 2.52	1 (2.0)	6 (16.7)	18 (30.5)	10 (71.4)	0.018	0.000	0.000	0.152	0.000	0.007
Hyperinsulinemia	0 (0)	1 (2.7)	1 (1.6)	4 (28.6)	0.416	1.000	0.001	1.000	0.017	0.004
Elevated blood pressure	6 (11.5)	16 (43.2)	28 (45.9)	10 (71.4)	0.001	0.000	0.000	0.837	0.116	0.137
BMI ≥ 25 kg/m^2^	0 (0)	30 (81.1)	61 (100)	14 (100)	0.000	0.000	0.000	0.001	0.169	NC
BMI ≥ 30 kg/m^2^	0 (0)	5 (13.5)	32 (52.5)	13 (92.9)	0.011	0.000	0.000	0.000	0.000	0.006
Abdominal obesity	0 (0)	7 (18.9)	61 (100)	14 (100)	0.001	0.000	0.000	0.000	0.000	NC
Excess visceral fat level	0 (0)	0 (0)	3 (4.9)	6 (42.9)	NC	0.248	0.000	0.293	0.000	0.001

In the general cohort, the structural and functional parameters of the heart were within reference intervals. However, the median LA volume, LV ESV, and LV myocardial mass significantly increased from groups 1 to 4, as evidenced by the Kruskal-Wallis test. According to the Kruskal-Wallis test, LV EF and SV index significantly decreased from group 1 to group 4. Significant differences were observed between groups 3 and 4 in LA volume, ESV, EF, CO, IVS thickness, and LV myocardial mass. Detailed characteristics of cardiac echocardiography are shown in Table 2.

**Table 2:** Characteristics of cardiac echocardiography in various groups Note: n: number of participants in a particular group; Me: median (interquartile range, 25th-75th percentile); p_1,2_: significance between group 1 and group 2; p_1,3_: significance between group 1 and group 3; p_1,4_: significance between group 1 and group 4; p_2,3_: significance between group 2 and group 3; p_2,4_: significance between group 2 and group 4; p_3,4_: significance between group 3 and group 4, calculated using the Mann‐Whitney test. p_K-W_: p-value from the Kruskal-Wallis test. LA: left atrium; LV: left ventricle; EDD: end-diastolic dimension; EDV: end-diastolic volume; ESD: end-systolic dimension; ESV: end-systolic volume; EF: ejection fraction; SV: stroke volume; CO: cardiac output.

Echo parameter	Normal BMI without AO and NAFLD (n = 52)	BMI ≥ 25 kg/m^2 ^or AO without NAFLD (n = 37)	BMI ≥ 25 kg/m^2 ^and AO without NAFLD (n = 61)	BMI ≥ 25 kg/m^2^, AO, and NAFLD (n = 14)	р₁,₂	р₁,₃	р₁,₄	р₂,₃	р₂,₄	р₃,₄	р_K-W_
	1	2	3	4							
	Me (25-75%)	Me (25-75%)	Me (25-75%)	Me (25-75%)							
LA volume, ml	46.0 (45.0-47.0)	47.0 (45.0-48.0)	48.0 (46.0-49.0)	49.0 (48.0-50.0)	0.221	0.001	0	0.083	0.003	0.034	0
LA dimension, cm	3.4 (3.4-3.5)	3.4 (3.4-3.6)	3.5 (3.4-3.6)	3.7 (3.4-3.8)	0.135	0	0.004	0.09	0.076	0.23	0.001
EDD, cm	4.3 (4.1-4.8)	4.6 (4.3-4.9)	4.6 (4.2-4.9)	4.9 (4.8-5.0)	0.076	0.005	0	0.369	0.002	0.016	0
EDV, ml	75.0 (68.5-78.0)	78.0 (69.0-81.0)	78.0 (78.0-87.0)	84.5 (78.0-89.0)	0.14	0	0	0.045	0.023	0.176	0
EDV index, cm/m^2^	42.1 (39.0-44.9)	40.2 (38.6-42.6)	40.5 (37.2-44.4)	39.4 (36.5-43.1)	0.093	0.363	0.06	0.642	0.486	0.196	0.207
ESD, cm	2.9 (2.8-2.9)	2.9 (2.7-3.0)	2.9 (2.8-3.0)	3.0 (2.8-3.0)	0.922	0.155	0.078	0.27	0.188	0.427	0.244
ESV, ml	27.3 (24.8-29.1)	28.5 (25.5-31.2)	30.4 (28.1-32.0)	33.8 (31.2-34.8)	0.071	0	0	0.061	0.011	0.049	0
ESV index, cm/m^2^	15.5 (14.1-16.7)	15.3 (14.3-16.1)	15.1 (14.0-17.4)	15.6 (14.0-16.9)	0.583	0.936	0.95	0.6	0.627	0.989	0.939
EF, %	63.0 (61.6-64.9)	62.0 (61.0-64.0)	62.0 (61.0-63.0)	60.0 (60.0-61.0)	0.144	0.004	0	0.304	0.003	0.008	0
SV, ml	46.9 (42.9-49.1)	46.8 (42.9-51.3)	49.0 (46.8-53.9)	50.7 (47.4-54.3)	0.546	0.001	0.006	0.054	0.069	0.678	0.003
SV index, ml/m^2^	26.5 (24.4-28.1)	24.6 (23.5-26.0)	25.3 (23.0-27.5)	23.8 (22.4-25.3)	0.009	0.151	0.001	0.422	0.06	0.037	0.005
CO, ml/min	3436.8 (2947.6-3835.3)	3310.0 (3074.4-3780.1)	3636.4 (3439.8-4163.3)	3867.3 (3472.0-4182.0)	0.927	0.004	0.013	0.02	0.023	0.554	0.004
End‐diastolic interventricular septal thickness (IVST), cm	0.8 (0.8-0.9)	0.9 (0.8-0.9)	0.9 (0.8-1.0)	1.0 (0.9-1.0)	0.015	0	0	0.147	0.024	0.075	0
End‐diastolic LV posterior wall thickness (PWT), cm	0.9 (0.8-1.0)	0.9 (0.8-1.0)	1.0 (0.9-1.0)	1.0 (0.9-1.0)	0.266	0	0.074	0.024	0.231	0.96	0.002
Relative wall thickness (RWT)	0.4 (0.4-0.4)	0.4 (0.4-0.4)	0.4 (0.4-0.4)	0.4 (0.4-0.4)	0.435	0.026	0.412	0.279	0.873	0.505	0.176
LV mass, gm	121.3 (102.5-135.2)	135.0 (122.5-165.5)	151.2 (118.4-173.7)	179.8 (151.3-179.8)	0.015	0	0	0.191	0.004	0.02	0
LV mass index (g/m^2^)	67.4 (61.3-77.2)	75.7 (61.0-87.1)	77.6 (62.6-89.8)	81.3 (72.7-84.7)	0.172	0.028	0.011	0.397	0.237	0.523	0.046

Even though all subjects from groups 3 and 4 had a BMI ≥ 25 kg/m^2^, a higher BMI was noted in group 4 (Me_3_ group: 30.2 (27.6-32.8) kg/m^2^ and Me_4_ group: 37.5 (35.2-39.4) kg/m^2^, p = 0.000). To assess the role of NAFLD independent of obesity, in the subsequent analyses, we adjusted the patients in these groups according to BMI. After adjustment, the BMI in these groups did not differ significantly (p = 0.114), detailed results are presented in Table 3. There were significant differences in the frequency of hypercholesterolemia between groups 1 and 2 compared with group 4. Hypertriglyceridemia was more common in group 4 than in group 1. Differences in the frequency of lower HDL-c levels remained only in groups 1 and 2. The frequency of increased LDL-c levels differed between groups 1 and 2 compared to groups 3 and 4. The increase in non-HDL-c levels was significantly higher in group 4 than in groups 1 and 2. The incidence of prediabetes was significantly lower in group 1 than in groups 2, 3, and 4. Homeostatic model assessment of insulin resistance (HOMA-IR) also increased from group 1 to group 4 but did not differ significantly between groups 3 and 4. At the same time, hyperinsulinemia was higher in group 4 than in all other groups, including group 3. The frequency of elevated blood pressure remained lower in group 1 than in the other groups.

**Table 3:** Characteristics of cardiometabolic risk factors in obese individuals with NAFLD after adjusting for BMI Note: n: number of participants; %: proportion of subjects as percent; p_1,2_: significance between group 1 and group 2; p_1,3_: significance between group 1 and group 3; p_1,4_: significance between group 1 and group 4; p_2,3_: significance between group 2 and group 3; p_2,4_: significance between group 2 and group 4; p_3,4_: significance between group 3 and group 4, calculated using chi-squared test. BMI: body mass index; AO: abdominal obesity; NAFLD: non-alcoholic fatty liver disease; HDL-c: high-density lipoprotein cholesterol; LDL-c: low-density lipoprotein cholesterol; non-HDL-c: non-high-density lipoprotein cholesterol; HOMA-IR: homeostatic model assessment of insulin resistance; NC: not calculated.

Parameter	Normal BMI without AO and NAFLD (n = 52)	BMI ≥ 25 kg/m^2^ or AO without NAFLD (n = 37)	BMI ≥ 25 kg/m^2^ and AO without NAFLD (n = 16)	BMI ≥ 25 kg/m^2^, AO, and NAFLD (n = 14)	р₁,₂	р₁,₃	р₁,₄	р₂,₃	р₂,₄	р₃,₄
	1	2	3	4						
	n (%)	n (%)	n (%)	n (%)						
Males	27 (51.9)	21 (56.8)	6 (37.5)	6 (42.9)	0.673	0.396	0.764	0.241	0.531	1.000
Females	25 (48.1)	16 (43.2)	10 (62.5)	8 (57.1)	0.673	0.396	0.764	0.241	0.531	1.000
Total cholesterol ≥ 5 mmol/L	16 (30.8)	10 (27.0)	8 (50.0)	9 (64.3)	0.814	0.231	0.031	0.125	0.023	0.484
Triglycerides ≥ 1.7 mmol/L	3 (5.8)	4 (10.8)	3 (18.8)	5 (35.7)	0.443	0.137	0.008	0.419	0.093	0.417
Low HDL-c (in males < 1.0 mmol/L; females < 1.2 mmol/L)	8 (15.4)	14 (37.8)	6 (37.5)	3 (21.4)	0.024	0.078	0.688	1.000	0.334	0.440
LDL-c > 3 mmol/L	23 (44.2)	15 (40.5)	12 (75.0)	13 (92.9)	0.829	0.045	0.002	0.035	0.001	0.336
Non-HDL-c > 3.4 mmol/L	20 (38.5)	15 (41.7)	10 (62.5)	12 (85.7)	0.826	0.149	0.002	0.232	0.010	0.226
Hyperuricemia	4 (7.7)	6 (16.2)	2 (12.5)	0 (0)	0.308	0.620	0.571	1.000	0.170	0.485
Prediabetes	3 (5.8)	8 (21.6)	5 (31.3)	7 (50.0)	0.046	0.015	0.002	0.499	0.166	0.707
HOMA-IR > 2.5	1 (2.0)	6 (16.7)	8 (50.0)	10 (71.4)	0.018	0.000	0.000	0.019	0.000	0.284
Hyperinsulinemia	0 (0)	1 (2.7)	0 (0)	4 (28.6)	0.416	NC	0.001	1.000	0.017	0.037
Elevated blood pressure	6 (11.5)	16 (43.2)	9 (56.3)	10 (71.4)	0.001	0.001	0.000	0.550	0.116	0.466
BMI ≥ 25 kg/m^2^	0 (0)	30 (81.1)	16 (100)	14 (100)	0.000	0.000	0.000	0.088	0.169	NC
BMI ≥ 30 kg/m^2^	0 (0)	5 (13.5)	16 (100)	13 (92.9)	0.011	0.000	0.000	0.000	0.000	0.467
Abdominal obesity	0 (0)	7 (18.9)	16 (100)	14 (100)	0.001	0.000	0.000	0.000	0.000	NC
Excess visceral fat level	0 (0)	0 (0)	3 (18.8)	6 (42.9)	NC	0.011	0.000	0.025	0.000	0.236

There was a statistically significant difference in the median values of insulin and HOMA-IR between groups 3 and 4. There was a trend toward an increase in the glycated hemoglobin levels from group 3 to group 4. The detailed analysis is presented in Table 4.

**Table 4:** Quantitative characteristics of lipid and carbohydrate metabolism in individuals with obesity and NAFLD after adjusting for BMI Note: n: number of participants in a particular group; Me: median (interquartile range, 25th-75th percentile); p_1,2_: significance between group 1 and group 2; p_1,3_: significance between group 1 and group 3; p_1,4_: significance between group 1 and group 4; p_2,3_: significance between group 2 and group 3; p_2,4_: significance between group 2 and group 4; p_3,4_: significance between group 3 and group 4, calculated using the Mann‐Whitney test. p_K-W_: p-value from the Kruskal-Wallis test. BMI: body mass index; AO: abdominal obesity; NAFLD: non-alcoholic fatty liver disease; HDL-c: high-density lipoprotein cholesterol; LDL-c: low-density lipoprotein cholesterol; non-HDL-c: non-high-density lipoprotein cholesterol; HOMA-IR: homeostatic model assessment of insulin resistance; HbA1c: glycated hemoglobin.

Parameter	Normal BMI without AO and NAFLD (n = 52)	BMI ≥ 25 kg/m^2^ or AO without NAFLD (n = 37)	BMI ≥ 25 kg/m^2 ^and AO without NAFLD (n = 16)	BMI ≥ 25 kg/m^2^, AO, and NAFLD (n = 14)	р_₁,₂_	р_₁,₃_	р_₁,₄_	р_₂,₃_	р_₂,₄_	р_₃,₄_	р_K-w_
	1	2	3	4							
	Me (25-75%)	Me (25-75%)	Me (25-75%)	Me (25-75%)							
Total cholesterol, mmol/L	4.5 (3.8-5.1)	4.4 (3.8-5.1)	5.0 (4.6-5.5)	5.6 (4.8-6.9)	0.963	0.039	0	0.036	0.001	0.142	0.001
Triglycerides, mmol/L	0.7 (0.6-0.9)	0.8 (0.6-1.1)	1.1 (0.9-1.5)	1.5 (1.3-2.5)	0.237	0	0	0.011	0.001	0.12	0
HDL-c, mmol/L	1.4 (1.2-1.6)	1.2 (1.0-1.5)	1.3 (1.0-1.5)	1.3 (1.2-1.5)	0.071	0.298	0.583	0.839	0.342	0.667	0.299
LDL-c, mmol/L	2.9 (2.3-3.5)	2.7 (2.4-3.4)	3.4 (2.9-3.9)	3.7 (3.4-4.8)	0.703	0.027	0	0.019	0	0.131	0
Non-HDL-c, mmol/L	3.2 (2.5-3.6)	3.0 (2.6-3.7)	3.7 (3.3-4.4)	4.3 (3.6-5.4)	0.59	0.009	0	0.027	0.001	0.12	0
Insulin, μIU/mL	5.4 (4.2-6.9)	8.4 (5.3-12.0)	11.9 (11.3-16.4)	24.2 (11.0-27.5)	0	0	0	0.018	0	0.043	0
HOMA-IR	1.0 (0.7-1.2)	1.6 (1.0-2.4)	2.4 (2.1-3.4)	4.9 (2.1-6.9)	0	0	0	0.014	0	0.038	0
HbA1c, %	5.2 (4.9-5.6)	5.4 (5.1-5.7)	5.5 (5.0-6.0)	5.9 (5.4-6.2)	0.088	0.221	0	0.69	0.009	0.077	0.003

A feature of group 4 was that NAFLD was associated with more pronounced obesity. Therefore, initially, in patients with NAFLD, the structural and functional parameters of the heart differed between the groups. However, after adjusting for BMI, the trend toward differences between groups 3 and 4 remained only for the left ventricle ejection fraction (LVEF) and the SV index (Table 5).

**Table 5:** Quantitative characteristics of cardiac echocardiographic parameters after adjusting for BMI Note: n: number of participants in a particular group; Me: median (interquartile range, 25th-75th percentile); p_1,2_: significance between group 1 and group 2; p_1,3_: significance between group 1 and group 3; p_1,4_: significance between group 1 and group 4; p_2,3_: significance between group 2 and group 3; p_2,4_: significance between group 2 and group 4; p_3,4_: significance between group 3 and group 4, calculated using the Mann‐Whitney test; p_K-W_: p-value from the Kruskal-Wallis test. BMI: body mass index; AO: abdominal obesity; NAFLD: non-alcoholic fatty liver disease; LA: left atrium; LV: left ventricle; EDD: end-diastolic dimension; EDV: end-diastolic volume; ESD: end-systolic dimension; ESV: end-systolic volume; EF: ejection fraction; SV: stroke volume; CO: cardiac output; IVST: end‐diastolic interventricular septal thickness; PWT: end‐diastolic LV posterior wall thickness; RWT: relative wall thickness.

Parameter	Normal BMI without AO and NAFLD (n = 52)	BMI ≥ 25 kg/m^2 ^or AO without NAFLD (n = 37)	BMI ≥ 25 kg/m^2 ^and AO without NAFLD(n = 16)	BMI ≥ 25 kg/m^2^, AO, and NAFLD (n = 14)	р_₁,₂_	р_₁,₃_	р_₁,₄_	р_₂,₃_	р_₂,₄_	р_₃,₄_	р_K-W_
	1	2	3	4							
	Me (25-75%)	Me (25-75%)	Me (25-75%)	Me (25-75%)							
LA volume, ml	46.0 (45.0-47.0)	47.0 (45.0-48.0)	49.0 (47.5-50.0)	49.0 (48.0-50.0)	0.221	0.001	0	0.013	0.003	0.822	0
LA dimension, cm	3.4 (3.4-3.5)	3.4 (3.4-3.6)	3.5 (3.5-3.7)	3.7 (3.4-3.8)	0.135	0.003	0.004	0.056	0.076	0.697	0.003
EDD, cm	4.3 (4.1-4.8)	4.6 (4.3-4.9)	4.9 (4.2-5.0)	4.9 (4.8-5.0)	0.076	0.011	0	0.117	0.002	0.498	0
EDV, ml	75.0 (68.5-78.0)	78.0 (69.0-81.0)	80.0 (78.0-91.0)	84.5 (78.0-89.0)	0.14	0	0	0.021	0.023	0.886	0
EDV index, cm/m^2^	42.1 (39.0-44.9)	40.2 (38.6-42.6)	41.1 (38.0-44.6)	39.4 (36.5-43.1)	0.093	0.37	0.06	0.816	0.486	0.377	0.171
ESD, cm	2.9 (2.8-2.9)	2.9 (2.7-3.0)	3.0 (2.9-3.1)	3.0 (2.8-3.0)	0.922	0.001	0.078	0.014	0.188	0.423	0.012
ESV, ml	27.3 (24.8-29.1)	28.5 (25.5-31.2)	30.4 (29.2-36.1)	33.8 (31.2-34.8)	0.071	0	0	0.04	0.011	0.525	0
ESV index, cm/m^2^	15.5 (14.1-16.7)	15.3 (14.3-16.1)	15.4 (13.7-17.6)	15.6 (14.0-16.9)	0.583	0.885	0.95	0.67	0.627	0.984	0.936
EF, %	63.0 (61.6-64.9)	62.0 (61.0-64.0)	61.0 (61.0-62.5)	60.0 (60.0-61.0)	0.144	0.013	0	0.236	0.003	0.07	0
SV, ml	46.9 (42.9-49.1)	46.8 (42.9-51.3)	49.9 (47.6-54.9)	50.7 (47.4-54.3)	0.546	0.001	0.006	0.035	0.069	0.525	0.003
SV index, ml/m^2^	26.5 (24.4-28.1)	24.6 (23.5-26.0)	25.4 (23.6-27.0)	23.8 (224-25.3)	0.009	0.129	0.001	0.561	0.06	0.077	0.002
CO, ml/min	3436.8 (2947.6-3835.3)	3310.0 (3074.4-3780.1)	3679.8 (3235.4-4434.7)	3867.3 (3472.0-4182.0)	0.927	0.043	0.013	0.102	0.023	0.822	0.026
IVST, cm	0.8 (0.8-0.9)	0.9 (0.8-0.9)	0.9 (0.8-1.0)	1.0 (0.9-1.0)	0.015	0.001	0	0.154	0.024	0.4	0
PWT, cm	0.8 (0.9-1.0)	0.9 (0.8-1.0)	1.0 (0.9-1.0)	1.0 (0.9-1.0)	0.266	0.01	0.074	0.097	0.231	0.854	0.038
RWT	0.4 (0.4-0.4)	0.4 (0.4-0.4)	0.4 (0.4-0.4)	0.4 (0.4-0.4)	0.435	0.17	0.412	0.478	0.873	0.552	0.524
LV mass, gm	121.3 (102.5-135.2)	135.6 (122.5-165.5)	162.4 (114.8-186.1)	179.8 (151.3-179.8)	0.015	0.002	0	0.09	0.004	0.58	0
LV mass index (g/m^2^)	67.4 (61.3-77.2)	75.7 (61.0-87.1)	80.3 (57.7-91.5)	81.3 (72.7-84.7)	0.172	0.118	0.011	0.383	0.237	0.984	0.064

After adjusting for BMI, we performed a correlation analysis to study the relationship between laboratory and instrumental parameters and indices of liver steatosis and fibrosis with cardiac structure and function (Table 6). ALT and AST levels are weakly correlated with LA volume, LV EDV, LV SV, and LV mass. The HSI values moderately correlated with LA volume, LV ESV and EDV, LV SV, and LV mass, and a weak inverse relationship with the LVEF and SV index. A moderate strength correlation was found between the TyG Index and LV EDV values, IVS thickness, and LV mass, and a weak relationship between the TyG index and LA volume, LV ESV, and SV. Significant correlations were established between CAP and LSM with LA volume, CAP with IVS thickness and LV mass, and an inverse relationship between CAP and the SV index of medium strength on the Chaddock scale. We found a medium-strength correlation between non-HDL-c and LV mass when analyzing the lipid parameters.

**Table 6:** Correlation of key echocardiographic measures with parameters of laboratory and instrumental test, indices of liver steatosis and fibrosis, as well as of lipid and carbohydrate metabolism Note: rs: Spearman’s correlation coefficient; p: statistical significance. ALT: alanine aminotransferase; AST: aspartate aminotransferase; NAFLD-LFS: non-alcoholic fatty liver disease-liver fat score; HSI: hepatic steatosis index; TyG index: triglyceride-glucose index; FIB-4: fibrosis-4; BMI: body mass index; CAP: controlled attenuation parameter; LSM: liver stiffness measurement; HDL-c: high-density lipoprotein cholesterol; LDL-c: low-density lipoprotein cholesterol; non-HDL-c: non-high-density lipoprotein cholesterol; HOMA-IR: homeostatic model assessment of insulin resistance; HbA1c: glycated hemoglobin; LA: left atrium; LV: left ventricle; EDV: end-diastolic volume; ESV: end-systolic volume; EF: ejection fraction; SV: stroke volume; IVST: end‐diastolic interventricular septal thickness; LVMI: left ventricle mass index; RWT: relative wall thickness; PWT: end‐diastolic LV posterior wall thickness.

Parameters		LA volume	EDV	ESV	EF	SV	SV index	IVST	LV mass	LVMI	RWT	PWT
ALT	r_s_	0.212	0.280	0.225	-0.089	0.286	-0.129	0.194	0.263	0.106	0.022	-0.031
	p	0.007	0.000	0.004	0.258	0.000	0.098	0.013	0.001	0.176	0.778	0.690
AST	r_s_	0.173	0.189	0.141	-0.028	0.196	-0.134	0.152	0.208	0.081	0.009	-0.057
	p	0.027	0.015	0.071	0.717	0.012	0.087	0.053	0.008	0.303	0.904	0.472
NAFLD-LFS	r_s_	0.209	0.203	0.235	-0.169	0.149	-0.188	0.160	0.202	0.037	-0.015	0.105
	p	0.008	0.009	0.003	0.031	0.057	0.016	0.041	0.010	0.643	0.849	0.181
HSI	r_s_	0.382	0.383	0.407	-0.227	0.313	-0.154	0.265	0.366	0.141	0.007	0.147
	p	0.000	0.000	0.000	0.004	0.000	0.049	0.001	0.000	0.072	0.928	0.061
TyG index	r_s_	0.192	0.320	0.296	-0.105	0.295	-0.134	0.316	0.333	0.162	0.129	0.157
	p	0.015	0.000	0.000	0.185	0.000	0.088	0.000	0.000	0.040	0.102	0.046
FIB-4	r_s_	-0.046	0.023	0.039	-0.035	0.006	0.164	0.040	0.016	0.064	0.004	-0.105
	p	0.562	0.775	0.625	0.656	0.936	0.052	0.613	0.839	0.417	0.961	0.182
BMI	r_s_	0.445	0.471	0.495	-0.334	0.361	-0.243	0.359	0.468	0.196	0.112	0.217
	p	0.000	0.000	0.000	0.000	0.000	0.002	0.000	0.000	0.012	0.153	0.005
САР	r_s_	0.532	0.433	0.415	-0.215	0.434	-0.512	0.540	0.651	0.246	-0.247	0.151
	p	0.034	0.082	0.098	0.408	0.081	0.036	0.025	0.005	0.342	0.340	0.562
LSM	r_s_	0.538	0.355	0.455	-0.179	0.319	-0.048	0.017	0.005	0.023	0.532	0.276
	p	0.031	0.162	0.067	0.492	0.211	0.855	0.948	0.984	0.932	0.028	0.284
Total cholesterol	r_s_	0.152	0.178	0.186	-0.120	0.147	-0.021	0.211	0.259	0.202	0.076	0.010
	p	0.054	0.023	0.017	0.127	0.060	0.785	0.007	0.001	0.009	0.332	0.903
Triglycerides	r_s_	0.225	0.290	0.277	-0.107	0.262	-0.132	0.298	0.320	0.148	0.091	0.108
	p	0.004	0.000	0.000	0.172	0.001	0.093	0.000	0.000	0.059	0.245	0.170
HDL-c	r_s_	-0.139	-0.165	-0.156	0057	-0.151	0.064	-0.164	-0.194	-0.104	0.015	-0.086
	p	0.079	0.035	0.046	0.470	0.054	0.418	0.036	0.013	0.183	0.847	0.274
LDL-c	r_s_	0.210	0.181	0.201	-0.148	0.140	-0.053	0.197	0.292	0.219	0.022	0.015
	p	0.008	0.021	0.010	0.060	0.075	0.503	0.012	0.000	0.005	0.777	0.845
Non-HDL-c	r_s_	0.198	0.229	0.237	-0.144	0.192	-0.061	0.269	0.331	0.241	0.062	0.038
	p	0.012	0.003	0.002	0.067	0.014	0.437	0.001	0.000	0.002	0.429	0.628
Insulin	r_s_	0.074	0.117	0.089	0.016	0.125	-0.036	0.165	0.121	0.047	-0.001	0.118
	p	0.350	0.135	0.257	0.836	0.110	0.651	0.034	0.123	0.552	0.987	0.133
HOMA-IR	r_s_	0.062	0.119	0.092	0.012	0.125	-0.034	0.156	0.118	0.049	-0.010	0.108
	p	0.435	0.134	0.245	0.884	0.116	0.672	0.049	0.137	0.540	0.903	0.175
HbA1c	r_s_	-0.043	-0.084	-0.050	-0.029	-0.102	-0.121	-0.048	-0.030	-0.044	-0.079	0.132
	p	0.586	0.283	0.527	0.715	0.195	0.122	0.540	0.704	0.578	0.316	0.093

To determine the cut-off values of cardiac structural and functional parameters in patients with NAFLD, we performed ROC analysis after adjusting the groups according to BMI (Table 7).

**Table 7:** Results of ROC analysis of echocardiography parameters in patients with NAFLD АUC: area under the curve; CI: 95% confidence interval; p: statistical significance; LA: left atrium; EDV: end-diastolic volume; ESV: end-systolic volume; EF: ejection fraction; SV: stroke volume; IVST: end‐diastolic interventricular septal thickness; LV: left ventricle; RWT: relative wall thickness; PWT: end‐diastolic LV posterior wall thickness; ROC: receiver operator characteristic.

Parameters	Cut-off	Sensitivity, %	Specificity, %	AUC, %	CI	p
LA volume, ml	47.5	78.6	64.2	75.7	0.627-0.888	0.001
EDV, ml	78.5	71.4	71.3	71.7	0.578-0.856	0.071
ESV, ml	31.1	78.6	74.0	75.8	0.628-0.889	0.067
EF, %	61.1	78.6	64.0	77.6	0.670-0.883	0.001
SV, ml	48.2	64.3	55.3	64.0	0.496-0.783	0.073
SV index, ml/m^2^	24.9	71.4	62.0	71.6	0.600-0.831	0.008
IVST, cm	0.9	78.6	44.7	72.9	0.592-0.865	0.005
LV mass, gm	151.3	78.6	66.7	78.0	0.662-0.897	0.001
LV mass index (g/m^2^)	79.5	71.4	67.3	62.7	0.483-0.771	0.117
RWT	0.4	64.3	49.3	50.5	0.373-0.637	0.948
PWT, cm	0.9	71.4	44.7	58.2	0.420-0.744	0.311

**Figure 2:**
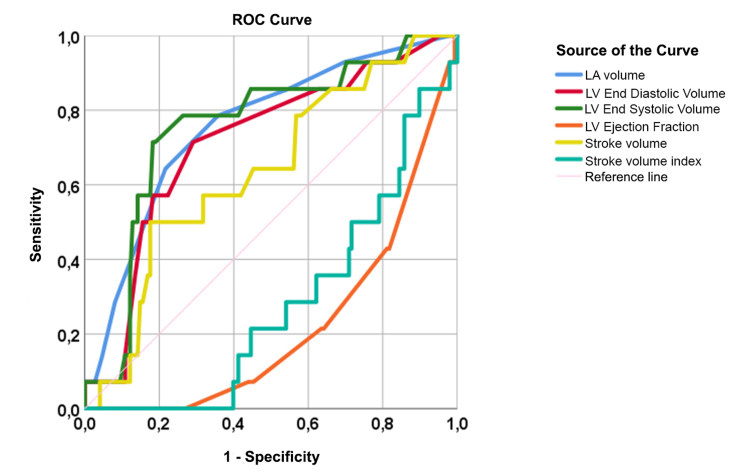
ROC curves for cardiac functional parameters in patients with NAFLD NAFLD: non-alcoholic fatty liver disease; LA: left atrium; LV: left ventricle; ROC: receiver operator characteristic.

A statistically significant model was obtained, showing an increase in LA volume and a decrease in LVEF and SV index. The ROC curves are shown in Figure 2.

**Figure 3:**
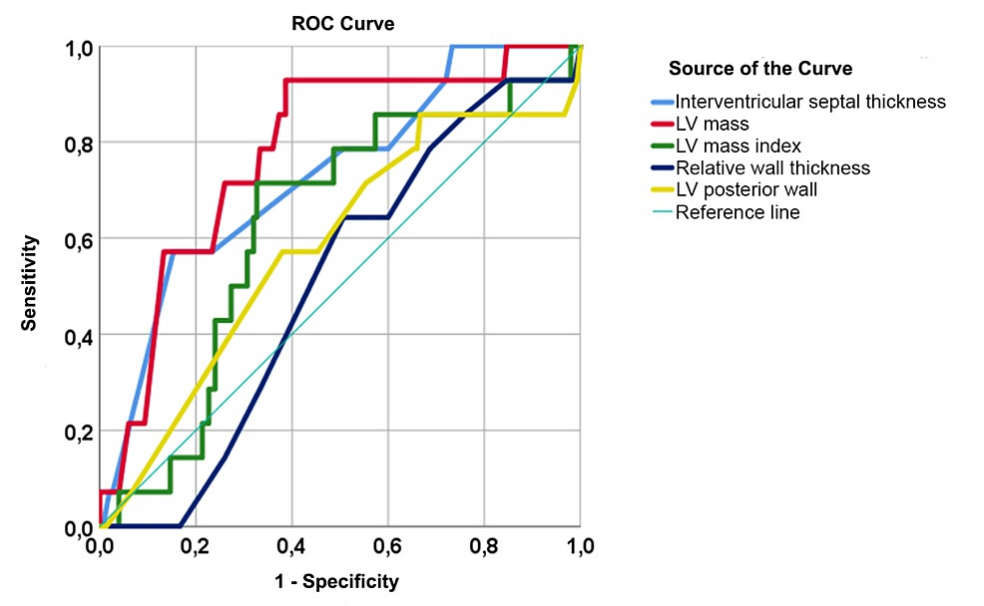
ROC curves for cardiac structural parameters in patients with NAFLD NAFLD: non-alcoholic fatty liver disease; LV: left ventricle; ROC: receiver operator characteristic.

Similarly, we performed a ROC analysis after adjusting for BMI to determine the cut-off values of structural heart parameters in patients with NAFLD. A statistically significant model of the increase in LV mass was obtained (Figure 3).

## Discussion

Among the studied young population, we observed a different profile of cardiometabolic risk factors in individuals with distinct obesity phenotypes [16]. When comparing groups with normal body weight, individuals with increased BMI and abdominal obesity (group 3), and a group with NAFLD (group 4), no additional changes in the risk factor profile were observed, except for the frequency of hyperinsulinemia and median insulin levels in NAFLD, which is consistent with the idea of the role of NAFLD in the regulation of carbohydrate metabolism [17]. Our data are consistent with the fact that a high incidence of hyperinsulinemia may, in turn, lead to stiffness of the cardiovascular system and cardiac remodeling [18-20].

In this study, we analyzed the association between NAFLD and echocardiographic parameters in patients without cardiometabolic disease. We identified and compared groups only with obesity and a combination of obesity and NAFLD. After adjusting for BMI, there was a trend toward a decrease in LVEF and SV index between groups of individuals with NAFLD. Our data reflect the general idea of the role of NAFLD in the development of heart failure with preserved ejection fraction (HFpEF). However, other studies have evaluated patients with T2DM, which makes it difficult to assess the independent contribution of NAFLD [21-24].

An additional argument in favor of the association of NAFLD with remodeling and cardiac hemodynamic changes is the results of correlations between calculated steatosis indices (HIS and TyG) as well as fibroelastometry parameters (САР) with echocardiographic parameters. These results are consistent with the more sensitive ROC analysis, where we determined the relationship of NAFLD with an increase in LA volume, IVS thickness, and LV mass, and a decrease in LVEF and LV SV index. Our results are in line with other research on the association of NAFLD with subclinical remodeling and myocardial dysfunction [25,26]. In obese individuals, the standard calculation of indices may not reflect an accurate picture [27], this may explain why the LV mass index did not show a significant relationship with NAFLD. However, we observed a significant relationship between LV myocardial mass and NAFLD, independent of obesity.

The strength of this study lies in the detailed clinical examination of the patients, laboratory and instrumental diagnostics, and a good sample size to obtain statistically significant results. We selected patients without cardiovascular diseases and diabetes, which allowed us to obtain data on the structural and functional changes in NAFLD and obesity. Our results presented data obtained using simple anthropometric methods to determine obesity. The liver steatosis and fibrosis indices used included the parameters available in clinical practice. Our findings may improve the effectiveness of a comprehensive assessment of patients and the development of strategies for primary and secondary prevention of HFpEF in NAFLD.

This study has some limitations. First, the design of a case-control study could theoretically lead to selection bias. Second, all patients with NAFLD were obese. Therefore, unambiguously speaking about the possible contribution of NAFLD, independent of obesity, to the structural and functional features of the heart is challenging. Finally, this was a one-off study, which limited the prospective estimates.

## Conclusions

Patients with NAFLD and obesity had a higher incidence of hyperinsulinemia and higher median insulin and HOMA-IR values. In individuals with NAFLD and obesity, an increase in LA volume, a decrease in LVEF and LV stroke volume index, and an increase in IVS thickness and LV mass were observed. The estimated steatosis index (HSI) is closely correlated with LA volume, ESV and EDV of LV, stroke volume, and LV mass. An association was established between an increase in the CAP score and an increase in LA volume, stroke volume index, IVS thickness, LV mass, and values of LSM with an increase in LA volume. Our results showed an association between NAFLD and the structural and functional parameters of the heart in individuals without cardiovascular disease and T2DM. The results of this study can also be used to improve the effectiveness of a comprehensive assessment of patients and to develop strategies for the primary and secondary prevention of HFpEF in NAFLD.
